# The need for cardiac surgery differential tariffs in Israel at the era of aging population and emerging technology: Importance of procedure type and patient complexity as assessed by EuroSCORE

**DOI:** 10.1186/s13584-021-00488-4

**Published:** 2021-09-06

**Authors:** J. Mendlovic, O. Merin, D. Fink, R. Tauber, E. Jacobzon, S. Tager, F. B. Mimouni, S. Silberman

**Affiliations:** 1 Hospital Management of Shaare Zedek Medical Center, affiliated with the Hadassah-Hebrew University School of Medicine, PO Box 3235, Jerusalem, Israel; 2 Department of Cardiothoracic Surgery, Shaare Zedek Medical Center, affiliated with the Hadassah-Hebrew University School of Medicine, Jerusalem, Israel; 3 Department of Neonatology, Sackler School of Medicine, Shaare Zedek Medical Center, Tel Aviv, Israel

**Keywords:** Cardiac surgery, PROCEDURE related group (PRG), EuroSCORE, Reimbursement, Cream skimming

## Abstract

**Background:**

Reimbursement for cardiac surgical procedures in Israel is uniform and does not account for diversity in costs of various procedures or for diversity in patient mix. In an era of new and costly technology coupled with higher risk patients needing more complex surgery, these tariffs may not adequately reflect the true financial burden on the caregivers. In the present study we attempt to determine whether case mix and complexity of procedures significantly affect cost to justify differential tariffs.

**Methods:**

We included all patients undergoing cardiac surgery at Shaare Zedek Medical Center between the years 1993–2016. Patients were stratified according to (1) type of surgery and (2) clinical profile as reflected by the predicted operative risk according to the European System for Cardiac Operative Risk Evaluation (EuroSCORE). Approximate cost of each group of patients was estimated by the average number of days in the Intensive Care Unit and days in the postoperative ward multiplied by the respective daily costs as determined by the Ministry of Health. We then added the fixed cost of the components used in the operating room (manpower and disposables). The final estimated cost (the outcome variable) was then evaluated as it relates to type of surgery and clinical profile. ANOVA was used to analyze cost variability between groups, and backward regression analysis to determine the respective effect of the abovementioned variables on cost. Because of non-normal distribution, both costs and lengths of stay were Log-transformed.

**Results:**

Altogether there were 5496 patients: 3863, 836, 685 and 112 in the isolated CABG, CABG + valve, 1 valve and 2 valves replacement groups. By ANOVA, the costs in all EuroSCORE subgroups were significantly different from each other, increasing with increased EuroSCORE subgroup. Cost was also significantly different among procedure groups, increasing from simple CABG to single valve surgery to CABG + valve surgery to 2-valve surgery. In backward stepwise multiple regression analysis, both type of procedure and EuroSCORE group significantly impacted cost. ICU stay and Ward stay were significantly but weakly related while EuroSCORE subgroup was highly predictive of both ICU stay and ward stay.

**Conclusions:**

The cost of performing heart surgery today is directly influenced by both patient profile as well as type of surgery, both of which can be quantified. Modern day technology is costly yet has become mandatory. Thus reimbursement for heart surgery should be based on differential criteria, namely clinical risk profile as well as type of surgery. Our results suggest an urgent need for design and implementation of a differential tariff model in the Israeli reimbursement system. We suggest that a model using a fixed, average price according to the type of procedure costs, in addition to a variable hospitalization cost (ICU + ward) determined by the patient EuroSCORE or EuroSCORE subgroup should enable an equitable reimbursement to hospitals, based on their case mix.

## Background

Cost of performing heart surgery is variable, and correlates with type of surgery as well as patient profile. However, reimbursement for cardiac surgery procedures in Israel today is uniform according to the Procedure Related Group (PRG). The terminology more commonly used, and which we will refer to, is the Procedure related group (PRG) reimbursement method, which we will refer to. Of note, the Israeli reimbursement method does not consider the diversity in costs of various procedures. The cost of surgery varies widely between patients, and correlates with length of hospitalization, as well as costs of individual patients such as imaging, dialysis etc. This may result in the preferential treatment of younger and healthier patients, and economic considerations might play a role in patient referral. We performed a retrospective analysis of 5500 patients undergoing cardiac surgery and found great variability in costs between high and low risk patients. This situation poses an increasing financial burden on public, non-profit health providers, who carry the greatest part of the burden of treating complex and chronic patients. Hence, the problem is further intensified in light of the constant rise in life expectancy coupled with an increasing risk profile of patients undergoing surgery.

The objective of this study was to suggest a model for cost stratification in heart surgery in an attempt to predict relative cost according to patient profile and type of surgical procedure.

## Methods

This is a retrospective cohort study which includes all patients undergoing cardiac surgery between the years 1993 and 2016 in one center (the Shaare Zedek Medical Center). We Included all consecutive patients undergoing isolated coronary artery bypass (CABG), isolated valve surgery and mixed surgery (CABG + valve surgery or double valve surgery with or without other concomitant procedures). Patients undergoing surgery of the aorta were not included in this study as they are priced separately. This research was approved by our Institutional ethics committee, who waived the need of individual patient consent.

Cost was evaluated in relation to two independent variables: (i) patient related based on the clinical profile as represented by the EuroSCORE, an arithmetic score which predicts risk of in-hospital mortality after major cardiac surgery [1] on a scale between 0 and 45, and (ii) type of surgery. Procedure related cost was based on a prior detailed examination of cost components in the operating room (unpublished) which was conducted for internal use (n = 5500).

The European system for cardiac operative risk evaluation (EuroSCORE) was established as a risk stratification system to help assessing the quality of cardiac surgical case [1]. In brief, 17 risk factors were weighted for the final scoring system: 9 were patient-related, 4 were derived from the preoperative cardiac status and 4 on the timing and nature of the operation. Each factor was given a score, and the additive score was used to give an approximation of the predicted mortality: low, medium and high risk. The maximal additive score is 45. The logistic score takes ito considersation interactions between the various risk factors to give a more accurate estimate of mortality (Table 1).

**Table 1:** EuroSCORE

	Definition	Score
*Patient related*		
Age	Per 5 years or part of over 60	1
Female gender		1
Chronic pulmonary disease		1
Extracardiac arteriopathy		2
Neurologic dysfunction		2
Previous cardiac surgery		3
Elevated creatinine		2
Active endocarditis		3
Critical preoperative state		3
*Cardiac related*		
Unstable angina		2
LV dysfunction		1
Recent MI		2
Pulmonary hypertension		2
*Operation related*		
Emergency		2
Other than isolated CABG		2
Surgery on thoracic aorta		3
Postinfarction septal rupture		4

http://www.euroscore.org/

m, male; f, female; n.d., not determined; pos., positive test result

### Operating room and hospitalization cost components

The direct cost, excluding hospitalization components, is derived from several factors: (i) Hourly cost of the operating room: use of electricity, consumable gases and heart-lung machine. (ii) Hourly cost of personnel: surgeons, anesthesiologists, nurses, technicians and support staff. (iii) Disposables, with emphasis on equipment pertinent to cardiac surgery such as oxygenators and heart-lung machine related equipment. (iv) implanted devices such as prosthetic heart valves .

Hospitalization cost was calculated by the number of days in the intensive care unit plus the number of days in the surgical ward multiplied by the respective daily reimbursement as determined by the Ministry of Health.

Each of the 4 groups (by type of surgery performed) was further subdivided into 4 sub-groups according to the calculated EuroSCORE (EuroSCORE 0–2, 3–5, 6–8, and > 8). Total cost of the 12 groups was assessed by the number of days in the intensive care and regular ward multiplied by the appropriate daily reimbursement. This cost was added to the “operation cost” mentioned above.

### Statistical methods

The Minitab Statistical Package, version 17 (Minitab, State College, PA) was used for analyses. Data were tested for normality and expressed as mean ± standard deviations, or median and range as requested. Since length of stay and hospital stay costs were not distributed normally, they were Log-transformed in the analyses performed. A Stepwise backward multiple regression analysis was used to determine the relative effect of type of procedure and of EuroSCORE grouping of the patients (independent variable) on Log-transformed final costs (dependent variable). A *P*-value of < 0.05 was considered significant. Analysis of means using ANOVA was used to determine differences in stay or cost by EuroSCORE group or by procedure type.

## Results

Altogether there were 5496 patients: isolated CABG, n = 3863; CABG + 1 valve replacement, n = 836; single valve replacement, n = 685; and double valve replacement, n = 112. Table 2 depicts all patients who had entire data on Euroscore, final costs and type of surgery.

**Table 2:** Correlation between EuroSCORE, final costs and type of surgery

EuroSCORE	N*	Mean ± SD	Median	Range
CABG	3863	6.3 ± 8.7	3.3	0.9–86.7
1-valve	671	10.1 ± 12.7	6.0	1.5–80.1
1-valve + CABG	835	15.0 ± 15.2	9.4	1.5–85.4
2-valves	109	7.8 ± 10.1	4.8	1.5–86.6

Final cost (NIS)	N*	Mean ± SD	Median	Range
CABG	3578	61,405 ± 52,706	48,109	17,926-1,005,622
1-valve	632	195,387 ± 117,745	72,152	26,875-1,313,627
1-valve + CABG	606	125,281 ± 119,604	87,206	29,584-1,074,895
2-valves	97	125,680 ± 105,456	95,749	46,360–650,008

N* relates to all patients who had no missing value of neither EuroSCORE nor type of surgery. N* relates to all patients who had no missing value of neither Final cost nor type of surgery.

**Fig. 1:**
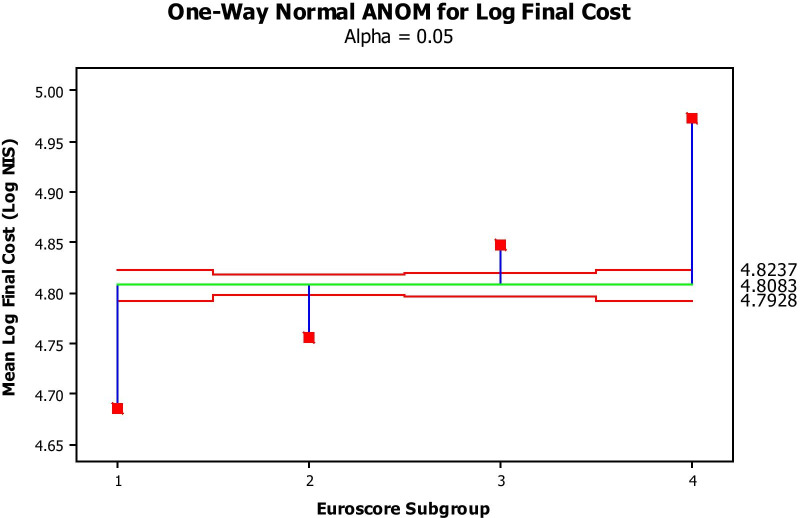
Mean Log final cost (Y axis, Log NIS) per Euroscore subgroup (1: 0–2; 2: 3–5; 3: 6–8; 4:>8). The green line is the average mean Log transformed final cost, while the 2 red lines are the 95% confidence limits. All values by Euroscore Subgroup fall outside the 95% confidence limits and therefore significantly differ from the average at a *P*-value of at least 0.05

**Fig. 2:**
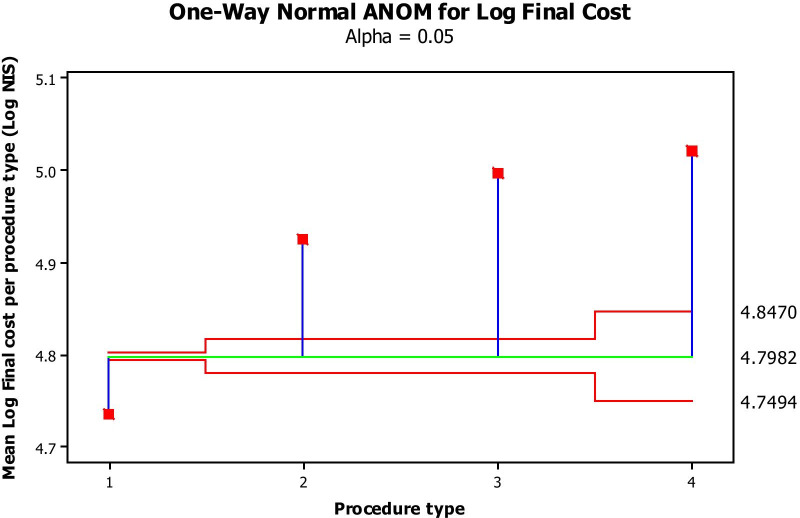
Mean Log transformed final cost (Y axis) per procedure (X axis: 1: CABG alone; 2: 1 valve surgery; 3: CABG + 1 valve; 4: 2-valves surgery). The green line is the average mean Log transformed final cost, while the 2 red lines are the 95% confidence limits. All values by procedure type fall outside the 95% confidence limits and therefore significantly differ from the average at a *P*-value of at least 0.05

**Fig. 3:**
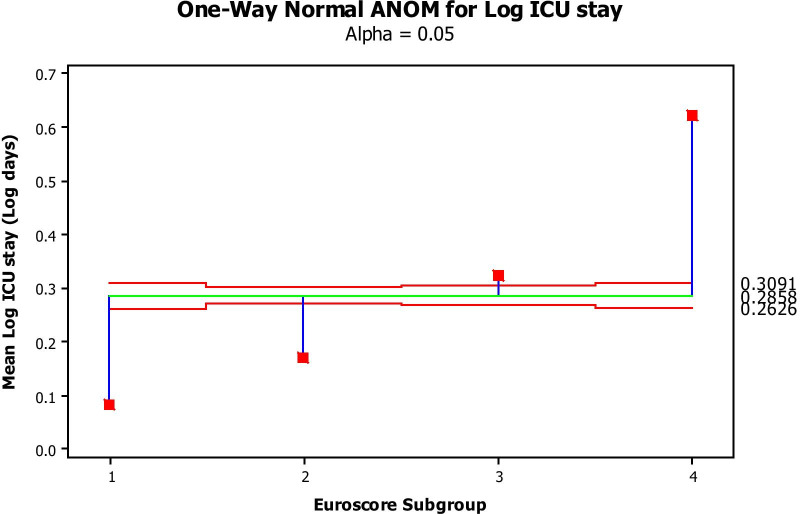
Mean Log transformed ICU stay (Y axis) per Euroscore group (X axis) (1: 0–2; 2: 3–5; 3: 6–8; 4:>8). The green line is the average mean Log transformed ICU stay, while the 2 red lines are the 95% confidence limits. All values by Euroscore Subgroup fall outside the 95% confidence limits and therefore significantly differ from the average at a *P*-value of at least 0.05

**Fig. 4:**
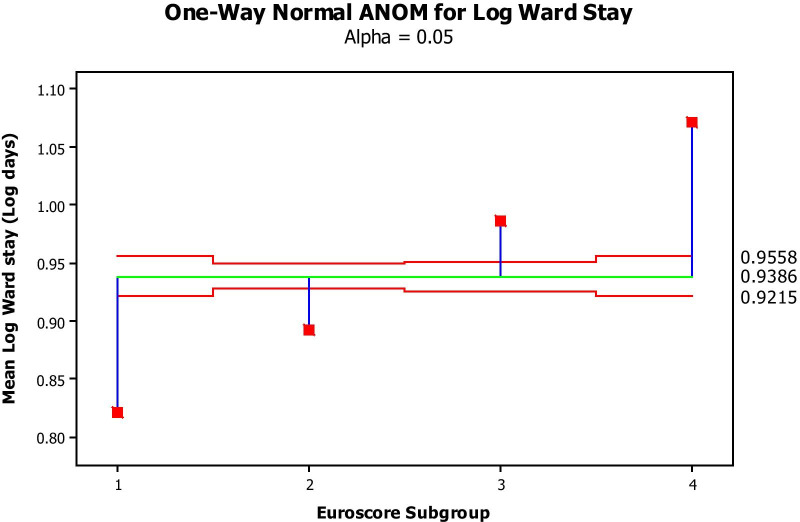
Mean Log ward stay (Y axis, Log days) per Euroscore subgroup (X axis: 1: 0–2; 2: 3–5; 3: 6–8; 4:>8). The green line is the average mean Log transformed ward stay, while the 2 red lines are the 95% confidence limits. All values by Euroscore Subgroup fall outside the 95% confidence limits and therefore significantly differ from the average at a *P*-value of at least 0.05

Log-transformed costs significantly increased as a function of increased predicted risk (Fig. 1) and surgical subgroups (Fig. 2). Log-transformed intensive care stay (Fig. 3) and general ward stay (Fig. 4) were both prolonged as a function of patient EuroSCORE.

**Fig. 5:**
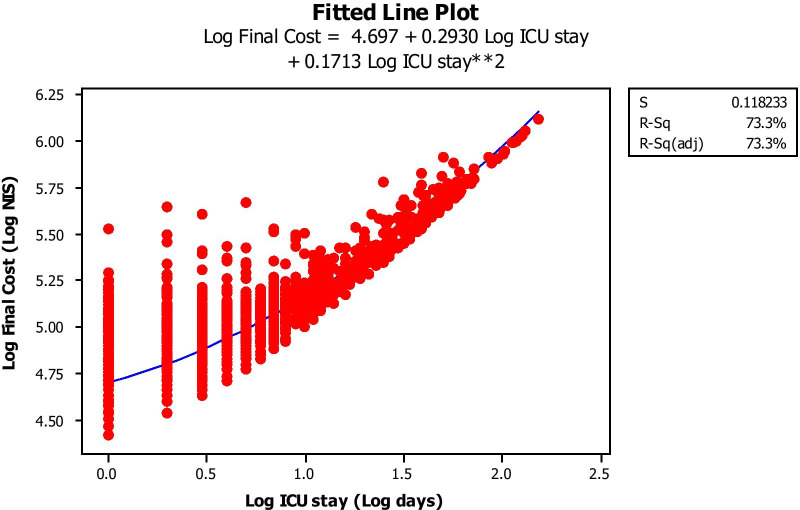
Log final cost (Y axis, Log NIS) as a function of ICU stay (X axis, Log days)

Figure 1 depicts the mean Log-transformed cost of surgery by EuroSCORE subgroup. The costs in all subgroups were significantly different from each other, with increasing cost with increasing EuroSCORE subgroup (ANOVA). Figure 2 depicts the average (Log-transformed) cost of each type of the procedures performed (analysis of means by ANOVA) This cost was significantly different between procedures, increasing from simple CABG to single valve replacement, while CABG + valve replacement and 2-valve replacement were the most, equally expensive procedures. Figure 3 shows that the higher the EuroSCORE subgroup, the higher the (Log-transformed) ICU stay (ANOVA). Figure 4 shows that the higher the EuroSCORE subgroup, the higher the Log transformed ward stay (ANOVA). In backward stepwise multiple regression analysis taking into account the Log-transformed final cost as the dependent variable, and type of procedure and EuroSCORE group as independent variables, both variables had a significant impact on Log-transformed cost (Model R^2^ = 28.8%, *P* < 0.001, partial R^2^ = 17.0% for EuroSCORE grouping and 11.8% for procedure type). Log-transformed ICU stay and Log-transformed Ward stay were significantly but weakly related (R^2^ = 11.2%, *P* < 0.05). The Log-transformed final cost was highly predicted by Log-transformed ICU stay (Fig. 5), (R^2^ = 70.7%, *P* < 0.001), and to a lesser extent by ward stay (R^2^ = 54.5%, *P* < 0.001).

## Discussion

Thousands of heart operations are performed each year in Israel. The most recent data are from 2018 to 2538 coronary bypass procedures, 1534 valve procedures, and 686 other heart operations performed annually in adults. This gives an estimate of approximately 4800 operations per year [2].

The current reimbursement (Procedure Related Group, or PRG), set by the Ministry of Health, for heart surgery is 79,000 NIS per case, regardless of the type of surgery, patient age and other comorbidities. During the last few years some changes have taken place in cardiac surgery tariffs. However, in spite of the fact that in 2013 major changes occurred in coding of heart surgery procedures, tariffs have changed by only 0.2% and no attempt was made to adjust tariffs to type of surgery and patient complexity. Thus, surgery involving only CABG will be reimbursed similar to surgery requiring CABG and replacement of two valves. It is obvious that the treating facility might face a financial loss in the latter case. With an estimate of 4800 operations per year these data indicate that the annual amount paid in Israel for heart surgery reaches nearly 379 million NIS a year. The current pricing system was established more than twenty years ago, and has not been modified other than for financial-related adjustments, such as the cost of living. It does not take into account the increasing complexity of current procedures and the patient profile, despite a marked effect of these on hospitalization costs. Changes in cost are derived from duration of hospitalization as well as from use of various resources [3–7]. On top of what is said, the technology used today in heart surgery is very advanced, and accordingly more expensive, yet has become mandatory, thus imposing an even greater financial burden on care providers.

Unlike the reimbursement method used in Israel, some western countries do not use a uniform pricing model [8]. In the U.S. for example, there is accurate pricing of hospitalization, namely billing according to type of surgery in addition to all hospitalization costs. These include rooming, additional procedures and counseling, disposables, medications etc. In the province of Ontario Canada on the other hand, hospitals receive a predetermined annual budget for cardiac surgery based on the estimated case load and regardless of the type of surgery. However, beyond that, it is the Ministry of Health that pays medical personnel individually for each procedure or consultation (“Fee for service”) so in effect salaries are not on the hospital’s budget (personal communication). Of note, some centers demand additional charge for items such as prosthetic valves. Even though the price of prosthetic valves comprises a significant part of the total PRG, the PRG in the public system remains uniform regardless of such additional costs.

The uniform pricing method used in Israel could lead to situations in which hospital management, both public and private, might give priority to patients whose overall expected cost will be low, thus depriving aging and other high-risk populations. A recently published comprehensive study by the OECD which refers to Israel’s aging population validates this potential [9]. The study, which was an important source of knowledge for the purpose of establishing a policy on population aging in Israel, showed that the proportion of people aged 75 and over in Israel is expected to rise by 50% over the next 20 years. A situation in which economic considerations might take precedence over medical decisions stands in complete contradiction to the aims of the Health Law which is “based on the principles of justice, equality and mutual assistance” [10]. There is a need for a reimbursement system that prevents discrimination of older patients and patients at high risk, and yet supports the health system that has the burden of treating them. This requires a differential model which takes into consideration the overall expenses of hospitalization and surgery by taking into account the most influential factors contributing to this cost, while using simple tools that do not require multiple resources. At present such a model is lacking.

Past studies have used mortality risk prediction models in order to estimate expectant costs by assuming a direct relationship between a patient’s chance of dying and the duration of hospitalization. Results of these studies indicate a direct relationship between risk of mortality and the duration of hospitalization [6, 7, 11–15]. The most prominent of these studies was conducted in 2004 and showed a clear correlation between EuroSCORE and hospitalization cost [12]. Most of these studies calculated overall cost assuming a constant daily price of hospitalization whether in the intensive care or the ward, and not by individual examination of all cost components. It should also be noted that these studies analyzed only patients undergoing coronary bypass surgery [7, 12]. Paradoxically, the very high-risk patients who die after a short hospitalization will cost the system less, but such an analysis and its ethical implications is beyond the scope of this study.

To build such a model, one needs first to understand the complex factors that affect the overall cost of hospitalization, which consists of two main parameters:

(i) The cost of the surgery itself, which varies depending on the type of surgery: “surgery components” and (ii) patient related factors such as age and comorbidity which may influence the need for unanticipated resource utilization. Our present study, while not offering an accurate cost estimate, shows that both above mentioned parameters affect overall cost, independently and in conjunction. Indeed, we found that the cost of surgery for a patient with a high EuroSCORE needing complex surgery is over three times the cost of simple coronary bypass on a young patient with no other co-morbidity (low EuroSCORE).

It is accepted to calculate hospitalization costs by summing the following components: personnel (medical, nursing, paramedical services, medical consultants), medication, supplies, imaging services and blood products [5]. These costs combine with the general overhead of the hospital, namely management salaries, cost of electricity etc. These are not affected directly by the volume of activity in the department. Overhead is usually calculated as 20% of total costs. In light of the great difficulty in testing all these factors, many studies have used cost per day in the ward and intensive care as a measure for determining the cost of hospitalization [3–7].

Several risk assessment models are available to aid in the decision-making process regarding referral to surgery. In our institution, we use customarily the EuroSCORE [16], thus we were able to use this tool in the present study. This model integrates the relative contribution of 17 parameters: age, gender, chronic lung disease, vascular disease, neurologic dysfunction, previous heart surgery, kidney failure, endocarditis, severe preoperative status, unstable angina, reduction in left ventricular function, recent myocardial infarction, pulmonary hypertension, emergency surgery, non-coronary bypass surgery, thoracic aorta surgery and ventricular septal rupture. The results are presented in an additive way so that young patients without risk factors have a score of 0 and those with the highest risk factors have a score of up to 45 [17]. The logistic calculation takes into account the relative impact of each risk factor and its interaction with all other risk factors and translates this to the estimated risk of operative death as a percentage. For simplicity we presented the additive result of the EuroSCORE.

### Limitations

Our study relies on calculated cost of operating room disposables performed in previous years and these have changed over time. Changes in practice patterns over time may substantially affect costs through the use of new technologies that may be cost-saving or on the contrary may increase costs. Additionally, since all patients were recruited from a single hospital, our results might not be necessarily applicable as such to other institutions. In addition, we did not calculate separately costs of patients who died versus costs of those who reached successful discharge. Finally, it is possible that some particular components of the Euroscore would be more predictive of costs than others, which would simplify the task of cost estimates. However, the Euroscore has been validated as a reliable predictor of mortality and major morbidities in other studies (REF).

## Conclusions

Our results show significant variations in the cost of hospitalization for heart surgery in Israel. There is up to threefold rise in cost between low risk and high risk patients. However, these results are based on the daily cost of hospitalization as determined by the Ministry of Health in the past, values which may not be pertinent to the present day. The existing economic state in Israel may not allow increased funding for cardiac surgery procedures. However, re-shuffling of finances within the system in accordance with patient mix might alleviate some of the excessive burden posed upon the treating facilities. We present the need of a financial model that will enable differential funding based on actual cost per procedure. This will remove potential financial incentives, prevent admission bias, and minimize financial losses for centers treating complex cases.

We suggest that a model using a fixed, average price according to the type of procedure costs, in addition to a variable hospitalization cost (ICU + ward) determined by the patient EuroSCORE or EuroSCORE subgroup should enable an equitable reimbursement to hospitals, based on their case mix. This model can be a potential benchmark for application to other procedural fields in medicine.

## Data Availability

Data is available.

## References

[1] Roques F, Michel P, Goldstone AR, Nashef SA (2003). The logistic EuroSCORE. Eur Heart J.

[2] http://www.health.gov.il/publicationsfiles/registrar_heart_surgery2018.pdf.

[3] Ferraris VA, Ferraris SP, Singh A (1998). Operative outcome and hospital cost. J Thorac Cardiovasc Surg.

[4] Mauldin PD, Weintraub WS, Becker ER (1994). Predicting hospital costs for first-time coronary artery bypass grafting from preoperative and postoperative variables. Am J Cardiol.

[5] Taylor GJ, Mikell FL, Moses HW, Dove JT, Katholi RE, Malik SA, Markwell SJ, Korsmeyer C, Schneider JA, Wellons HA (1990). Determinants of hospital charges for coronary artery bypass surgery: The economic consequences of postoperative complications. Am J Cardiol.

[6] Smith LR, Milano CA, Molter BS, Eleery JR, Sabiston DC, Smith PK (1994). Preoperative determinants of postoperative costs associated with coronary artery bypass graft surgery. Circulation.

[7] Riordan CJ, Engoren M, Zacharias A, Schwann TA, Parenteau GL, Durham SJ, Habib RH (2000). Resource utilization in coronary artery bypass operation: Does surgical risk predict cost?. Ann Thorac Surg.

[8] Gaughan J, Kobel C (2014). Coronary artery bypass grafts and diagnosis related groups: patient classification and hospital reimbursement in 10 European countries. Health Econ Rev.

[9] The Israel national institute for. health policy research annual report (The Dead Sea 2011 annual conference).

[10] *State Health Insurance Law, 1994.*.

[11] Geissler HJ, Holz P, Marohl S, Kuhn-Regnier F, Mehlhorn U, Sudkamp M, Rainer de Vivie E (2000). Risk stratification in heart surgery: comparison of six score systems. Eur J Cardio-thorac Surg.

[12] Nilsson J, Algotsson L, Hoglund P, Luhrs C, Brandt J (2004). EuroSCORE predicts intensive care unit stay and costs of open heart surgery. Ann Thorac Surg.

[13] Kurki TS, Hakkinen U, Lauharanta J, Ramo J, Leijala M (2002). Evaluation of the relationship between preoperative risk scores, postoperative and total length of stays and hospital costs in coronary bypass surgery. Eur J Cardio-thorac Surg.

[14] Kurki TS, Kataja MJ, Reich DL (2002). Validation of a preoperative risk index as a predictor of perioperative morbidity and hospital costs in coronary artery bypass graft surgery. J Cardiothorac Vasc Anesth.

[15] Sokolovic E, Schmidlin D, Schmid ER, Turina M, Ruef C, Schwenkglenks M, Szucs TD (2002). Determinants of costs and resource utilization associated with open heart surgery. Eur Heart J.

[16] http://www.euroscore.org/calc.html.

[17] Roques F, Nashef SA, Michel p, Gauducheau E, de Vincentiis C, Baudet E, Cortina J, David M, Faichney A, Gabrielle F, Gams E, Harjuk A, Jones MT, Pintor PP, Salamon R, Thulin L (1999). Risk factors and outcome in European cardiac surgery: analysis of the EuroSCORE multinational database of 19030 patients. Eur J Cardio-thorac Surg.

